# Consensus-Based Recommendations for the Diagnosis, Treatment, and Monitoring of Hypoparathyroidism: Insights from the DACH Region

**DOI:** 10.1007/s00223-025-01414-5

**Published:** 2025-08-12

**Authors:** Elena Tsourdi, Karin Amrein, Christian Meier, Markus Ketteler, Michael C. Kreissl, Annie Mathew, Tobias Vogelmann, Tino Schubert, Heide Siggelkow

**Affiliations:** 1 Department of Medicine III and Center for Healthy Aging, Technische Universität Dresden, Dresden, Germany; 2 Division of Endocrinology and Diabetology, Department of Internal Medicine, Medical University of Graz, Graz, Austria; 3 Division of Endocrinology, Diabetes & Metabolism, University Hospital Basel, Basel, Switzerland; 4 Department of General Internal Medicine and Nephrology, Robert-Bosch-Krankenhaus GmbH, Stuttgart, Germany; 5 Division of Nuclear Medicine, Department of Radiology and Nuclear Medicine, University Hospital Magdeburg, Otto-Von-Guericke University, Magdeburg, Germany; 6 Department of Endocrinology, Diabetes and Metabolism and Division of Laboratory Research, Endocrine Tumor Center at WTZ/ Comprehensive Cancer Center and ENETS Center of Excellence, University Hospital Essen, University of Duisburg-Essen, Essen, Germany; 7 LinkCare GmbH, Ludwigsburg, Germany; 8 Department of Trauma, Orthopedics and Reconstructive Surgery, Research and Development, University Medical Center Göttingen, Göttingen, Germany; 9 MVZ Endokrinologikum Göttingen, Göttingen, Germany

**Keywords:** Hypoparathyroidism, Parathyroid hormone, Hypocalcemia, Endocrine disorder, Consensus statements, Quality of life monitoring

## Abstract

Hypoparathyroidism (HypoPT) is a rare endocrine disorder characterized by low parathyroid hormone (PTH) levels, hypocalcemia, hyperphosphatemia, reduced active vitamin D (1,25-OH2 vitamin D), and hypercalciuria. Due to its rarity, non-specialized physicians often lack experience managing HypoPT. To address this, expert consensus statements were developed for the DACH region (Germany, Austria, Switzerland), considering regional differences and high HypoPT incidence. These statements aim to enhance adherence to guideline recommendations and improve non-specialist knowledge. From December 2023 to April 2024, three rounds of a Delphi consensus survey were conducted with seven DACH-region clinical experts. Consensus was defined as agreement among at least 6 of 7 participants (85%). Experts agreed surgery accounts for 90% of chronic HypoPT cases. Common symptoms include paresthesia, muscle cramps, and fatigue. Albumin-adjusted serum calcium should be measured 12–24 h post-surgically, within 2 weeks, and every 3–6 months thereafter. Key treatment goals are maintaining albumin-adjusted serum calcium in the lower normal range, symptom control, and quality of life. Long-term objectives include avoiding hypo- and hypercalcemia phases and disease-related complications. Failure of calcium and active vitamin D therapy is defined by persistent symptoms, hospitalization, laboratory values outside of the normal range, or medication intolerance. Experts emphasized using HypoPT-specific, validated quality-of-life questionnaires. This consensus provides practical guidance for non-specialists in diagnosing, treating, and monitoring HypoPT, improving care in German-speaking regions.

**Supplementary Information:**

The online version contains supplementary material available at 10.1007/s00223-025-01414-5.

## Introduction

Hypoparathyroidism (HypoPT) is an endocrine disorder characterized by decreased secretion of parathyroid hormone (PTH) and the biochemical constellation of hypocalcemia and hyperphosphatemia, reduced levels of active vitamin D (1,25-OH2 vitamin D), and hypercalciuria [1]. Care of patients suffering from HypoPT requires special expertise and experience to ensure treatment success, which is often lacking in practice [2, 3]. With an estimated prevalence of six to 34 affected persons per 100,000 inhabitants, HypoPT can be considered an orphan disease [4–9]. The annual incidence of HypoPT is estimated to be 0.80/100,000 inhabitants in Denmark [9] and 2.6/100,000 in India [10]. Regional differences exist with regards to epidemiology, etiology, diagnostic and therapeutic strategies [11]. In particular, thyroid surgery is one of the more common surgical procedures in Germany. In contrast to the USA and England, the number of thyroid surgeries in Germany is declining, however with approximately 109/100,000/year in 2012 is still elevated (Netherlands: 16/100,000/year, USA: at least 42/100,000/year, England: at least 27/100,000/year) [8]. Contributing factors include iodine deficiency, the frequent use of advanced diagnostics such as ultrasound, insufficient use of preoperative diagnostic measures such as fine needle biopsy, and the practice of "defensive medicine".

By far the most common cause of chronic HypoPT is inadvertent damage or removal of the parathyroid glands during anterior neck surgery (postsurgical HypoPT) [12]. In short term, HypoPT is associated with neuromuscular symptoms, seizures, cognitive and neuropsychiatric disorders and infections [13]. Long-term complications include cataracts, intracerebral calcifications, renal dysfunction and kidney stones, cataracts, cardiac arrhythmia and ischemic heart disease, depression, and increased mortality [14]. Measurement of serum calcium adjusted for albumin or ionized calcium and serum PTH concentrations is necessary to diagnose HypoPT [2, 15, 16].

The remaining cases of chronic HypoPT result from non-surgical etiologies, including autoimmune destruction of the parathyroid glands (either isolated or as part of autoimmune polyglandular syndrome type 1), congenital, other genetic disorders affecting parathyroid development or function, infiltrative diseases (for example, copper deposition in Wilson’s disease or iron overload in hemochromatosis), and direct gland damage. Severe metabolic disturbances such as profound magnesium excess or deficiency can also cause a transient, reversible hypoparathyroid state by suppressing parathyroid hormone secretion, and some patients have no identifiable cause [17].

Previous reports suggest that postsurgical etiology varies from 75% to nearly 90% of HypoPT patients [2, 17–19] and depends on the extent of the surgery and the surgeons’ expertise [7]. In German speaking countries in particular, thyroid surgeries are significantly more common than in other countries, suggesting a higher incidence of HypoPT, which emphasizes the importance of early diagnosis and management [8]. Whether HypoPT is considered acute and/or transient or chronic and permanent depends on the proximity of the surgical procedure to the onset of hypocalcemia and the duration of hypocalcemia. The majority of patients with acute HypoPT recover during the first 6–12 months [20]. If HypoPT persists over a prolonged postoperative period, it is referred to as chronic HypoPT. However, existing clinical guidelines differ with regard to the duration of this postoperative period, defining it between 6 and 12 months with no consensus so far on the exact definition [21, 22].

Several studies have investigated the precipitating factors for postoperative HypoPT. A recent meta-analysis identified 19 risk factors associated with HypoPT after thyroidectomy from 93 studies. Factors significantly linked with transient and permanent HypoPT include sex (female vs. male), cN stage, central neck dissection, lateral neck dissection, the extent of central neck dissection (bilateral vs. unilateral), type of surgery (total thyroidectomy [TT] vs. lobectomy), type of total thyroidectomy (TT vs. sub-TT), incidental parathyroidectomy, and pathology (cancer vs. benign) [23]. These factors should be taken into consideration with regards to a higher frequency of biochemical monitoring post-surgically.

Once diagnosed, treatment options include calcium supplements, native and active vitamin D with or without the addition of magnesium [24]. Treatment goals include serum albumin-adjusted calcium concentrations in the lower reference range, lack of symptoms, improved quality of life, defined as reduction of negative impact of physical, mental or emotional health, urinary calcium, serum phosphate, serum magnesium within reference ranges, and an adequate 25 OH vitamin D status (> 50 nmol/L) [21, 22]. Treatment success is monitored by serum measurements of albumin-adjusted calcium, phosphate, magnesium, creatinine, and 24-h urine measurement of calcium [21, 22]. In recent years, parathyroid hormone (PTH) was investigated as a new treatment option with currently two approved therapies in Europe; rhPTH(1–84) and palopegteriparatide. rhPTH(1–84) had been approved as an adjunctive treatment of adult patients with chronic hypoparathyroidism who cannot be adequately controlled with standard therapy alone—however, production of rhPTH(1–84) was terminated in 2024. Palopegteriparatide is currently used as a PTH replacement therapy in adults with chronic hypoparathyroidism [25, 26].

Regional guideline recommendations for diagnosis, treatment, and monitoring of HypoPT are limited. The only guideline in German language that includes recommendations for the treatment of HypoPT entitled “Operative Therapy of Benign Thyroid Diseases”, was drafted by experts from various specialist societies for endocrinology, diabetology, nuclear medicine, pathology, and thyroid diseases. It applies a surgical focus and therefore contains mainly immediate post-operative recommendations for HypoPT [27]. However, HypoPT is classified as chronic if it is still present at least 6 months following surgery. Therefore, the German-language guideline does not cover the long-term treatment for a persistent disease. International guidelines including Khan et al. [22] and Bollerslev et al. [21] are available. These consist of recommendations for the management of HypoPT beyond the postoperative period. However, the European and international guidelines are at some points inconsistent in their statements and not all statements are clearly operationalized for non-specialists. Examples of the inconsistencies of existing guidelines include the length of time regarding the classification of a HypoPT as chronic and/or the unspecific description of symptoms and treatment goals [21, 22]. As no specific recommendations are available in some areas due to lack of data or different approaches across countries, information on management is lacking, especially for medical non-specialized physicians.

Therefore, the aim of the present Delphi survey was to develop consensus statements on the diagnosis, treatment, and monitoring of HypoPT based on the opinions of leading experts in the field of HypoPT, with a focus on the DACH region taking into account regional aspects. The aim was to identify areas of agreement and disagreement among medical experts and to provide consensus recommendations for other healthcare professionals.

## Materials and Methods

### Study Design

**Fig. 1:**
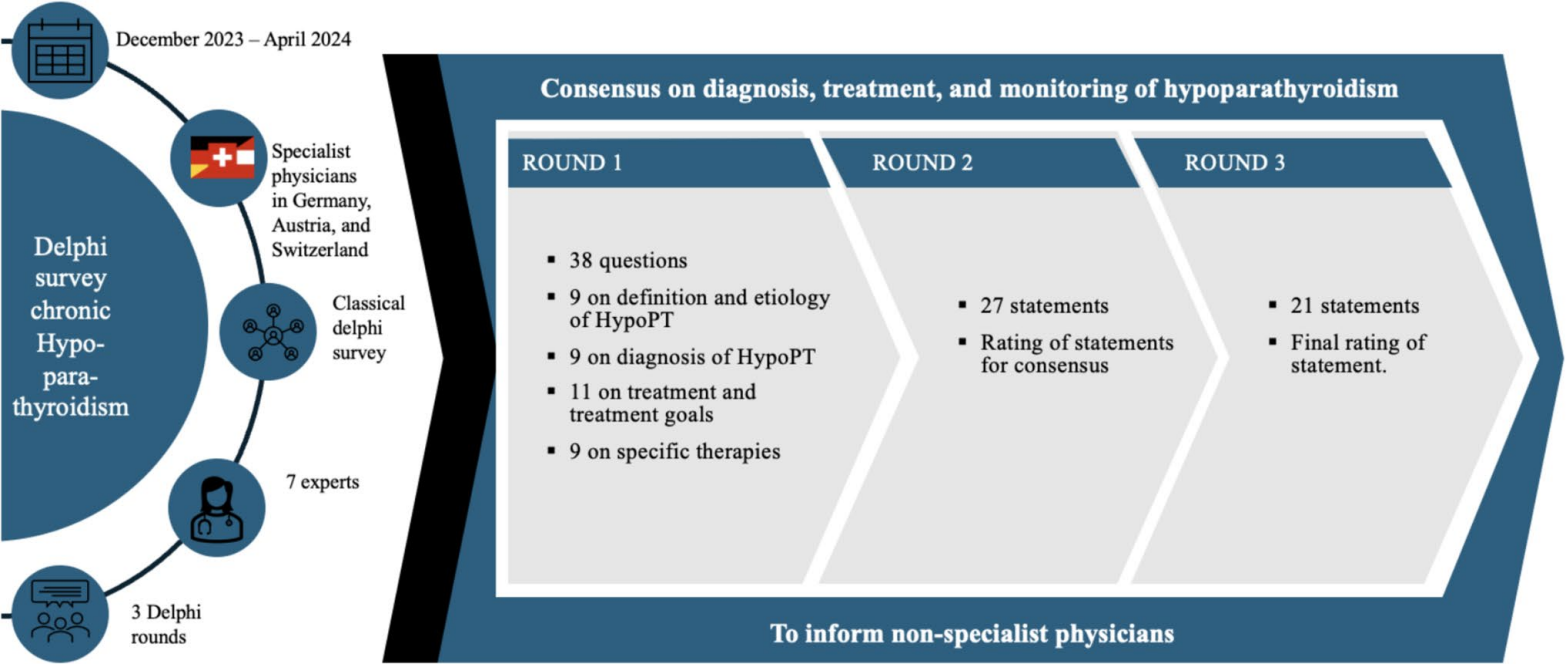
Outline of the study design

A classic Delphi survey with three rounds and consensus building [28] was conducted with seven German, Swiss and Austrian hypoparathyroidism experts between December 2023 and April 2024. An online questionnaire was used with the aim of identifying areas of treatment in which experts agree on how to diagnose, treat, and monitor patients in everyday medical practice (Fig. 1).

To ensure expert status, participants had to fulfill the following three requirements: First, they had to have published medical papers on HypoPT as lead or last author. Second, they had to be currently treating patients with HypoPT in their outpatient clinics and finally, they had to hold a leading position in clinical science and national/international professional societies.

Experts were invited to participate in the Delphi survey via e-mail and joined an initial kickoff meeting in Frankfurt, Germany in October 2023. The investigators at LinkCare GmbH, an independent research company, created round-based online questionnaires. The answers of the individual expert remained anonymous to each other throughout the entire study process. Results of the first round were summarized by the investigators and provided as feedback to the participants in the second Delphi round together with the revised questionnaire. To achieve consensus, the results of the second Delphi round were processed in the same way and presented to the experts in a third round.

### Questionnaires for the Three Delphi Rounds and Consensus Building

Following the initial meeting in Frankfurt, Germany, round-based online questionnaires pertaining the etiology, diagnosis, clinical manifestations, treatment and monitoring of chronic HypoPT were created by investigators at LinkCare GmbH (Ludwigsburg, Germany), an independent research company. Statements of international Consensus Papers were used as orientating documents, although this was not exhaustive. In particular, novel aspects such as PTH replacement therapy, which were not included in previous Consensus Papers, were highlighted in the current Delphi panel.

The questionnaire for the first Delphi round consisted of 38 questions divided into four question chapters. The first two questions of the first chapter of questions addressed the expert status of the respondents; all other questions were directly related to HypoPT. In addition to the expert status of the participants, the first set of questions focused on the definition and etiology of HypoPT and comprised 9 questions. The second set of questions also comprised 9 questions on diagnostics of HypoPT. Following this, the experts answered 11 questions on therapy with calcium and active vitamin D and the first Delphi round was completed with nine questions on specific therapies.

After completing the first round of the questionnaire, the answers were analyzed and aggregated. This was performed by calculating pre-defined statistical key figures, i.e., mean value, median, and distribution. The questionnaire was adapted for the second and third rounds to obtain consensus on the statements and assessments made by the experts in the first round.

The answers were to be given either numerically, or as free text or on a five-point Likert scale. In the Likert scales, the value “1” corresponds to “strongly disagree”, “2” to “disagree”, “3” to “neither”, “4” to “agree” and “5” to “strongly agree”. In the numerical estimation tasks, the feedback information provided to the experts contained information on the distribution of responses, which was represented by a histogram.

In the free-text tasks, the answers from the first round were reproduced and had to be ranked by the experts in the second round, according to their relevance. For the third Delphi round, the most frequent answers were formulated as statements and transferred to the five-point Likert scale to ask the experts for their agreement. Questions that were answered using the five-point Likert scale were used to build consensus.

Consensus was predefined, i.e., at least 85% of the experts (6 out of 7) had to answer with at least a “4” (corresponding to “agree”) or “5” (corresponding to fully agree) on a five-point Likert scale.[29, 30]. Statements were no consensus could be reached can be found in the Supplemental Materials, Table S1.

### Data Analysis

Descriptive statistics (median, minimum, maximum) were used to check for consensus between the different Delphi rounds. In the second and third round anonymous feedback from previous rounds in form of medians and histograms was given to the experts. All data were analyzed using Microsoft Excel 2019 16.4, Microsoft Corporation, Redmond (Washington), USA.

## Results

### Delphi Experts

By December 2023, seven out of ten invited HypoPT experts had agreed to participate. Six out of seven experts answered the first-round questionnaire and all seven out of seven answered the second and third round questionnaires. Of the 6 experts from the first round, 3 had more than 20 years of experience in the treatment of HypoPT. 2 of them have 10–19 years of experience and 1 expert had less than 10 years of experience. The average number of years of treatment experience with HypoPT was 16 years (median: 17 years). The experts were treating an average of 74 patients with HypoPT per year (median: 50 patients).

### Etiology and Diagnoses

The consensus of the experts was that surgery is the main cause (at least 90%) of chronic HypoPT (Table 1).

**Table 1:** Summary of statements on the etiology and diagnosis

Statement	Consensus
*Etiology and diagnoses*	
1. In at least 90% of all chronic hypoPT, surgery is the cause	↑↑
2. Paresthesia, muscle cramps and fatigue are the most common symptoms of HypoPT	↑↑
3. Albumin-adjusted serum calcium measurement for diagnosis can be performed 12–24 h post-surgically, within 2 weeks postoperatively and every 3–6 months thereafter	↑
4. The diagnosis requires a combination of serum albumin-adjusted calcium and serum parathyroid hormone levels	↑↑
5. There are no generally recognized threshold values for parathyroid hormone levels	↑

↑↑: 100% agreement, ↑: agreement > 85%

No consensus was reached on whether a surgically induced HypoPT will recover after 6 months (5 of 7 agreed). On the other hand, there was a consensus (6 out of 7) that a surgically induced HypoPT can no longer be expected to recover after 12 months.

Regarding the symptoms of HypoPT, all experts stated that paresthesia, muscle cramps and fatigue were the three most common manifestations (7 out of 7 agreed).

The experts also unanimously agreed on a combination of serum albumin-adjusted calcium, serum albumin, and serum PTH levels as a prerequisite for the diagnosis of HypoPT, whereas the measurement of isolated serum calcium and serum concentrations of 25 (OH) vitamin D is not adequate for diagnosis. Regarding serum albumin-adjusted calcium, they agreed on the possibility of an assessment 12–24 h post-surgically, within 2 weeks postoperative, as well as subsequently every 3 to 6 months thereafter. With regards to serum PTH levels, respondents agreed that there is no generally recognized threshold that could be used as a diagnostic criterion (6 out of 7 agreed). All respondents agreed that hypocalcemia should be further investigated.

The experts also agreed that thyroid ultrasound should be performed if necessary (6 out of 7 agreed).

### Treatment

The respondents uniformly agreed that the three most important short-term therapeutic goals in the treatment of chronic HypoPT were: serum albumin-adjusted calcium in the normal range, lack of symptoms and quality of life equivalent to age-and-sex-matched controls. According to the experts, the most important long-term goals are the avoidance of complications and the prevention of hypo- and hypercalcemia. These were confirmed by all 7 experts (Table 2).

**Table 2:** Summary of statements on the treatment

Statement	Consensus
*Treatment*	
6. Serum albumin-adjusted calcium in the lower normal range, freedom from symptoms and quality of life equivalent to age and gender controls are the most important short-term goals in chronic HypoPT	↑↑
7. Avoiding hypo- and hypercalcemia and preventing complications are the most important long-term goals in the treatment of chronic HypoPT	↑↑
8. The four most common reasons for the failure of therapy with calcium and active vitamin D are: Persistent symptoms and/or need for hospitalization Serum albumin-adjusted calcium levels outside the target range Intolerance to current treatment with calcium and active vitamin D Urine calcium outside the target range	↑

↑↑: 100% agreement, ↑: agreement > 85%

Experts estimated that on average, in 10% (with a range of 5 to 20%) of patients therapy with calcium and active vitamin D is inadequate and they require PTH replacement therapy. The four most common reasons for inadequate treatment with calcium and active vitamin D, as agreed by the experts, were persistent symptoms and/or need for hospitalization (57%), serum albumin-adjusted calcium levels or biochemical values out of normal range (72%), intolerance to treatment with calcium and active vitamin D (43%), and laboratory parameters such as urinary calcium (43%) out of target range.

### Monitoring

The experts unanimously supported the need to measure urinary calcium/creatinine levels at least once a year and unanimously agreed that assessing quality of life using validated questionnaires is relevant for monitoring therapy. All experts also agreed that quality of life questionnaires specifically designed and validated for HypoPT are important for this purpose (Table 3).

**Table 3:** Summary of statements on the monitoring

Statement	Consensus
*Monitoring*	
9. Quality of life measurements using validated questionnaires are relevant for therapy monitoring	↑↑
10. For quality of life measurements in HypoPT, a questionnaire specifically designed and validated for the disease would be important	↑↑
11. Urine calcium/creatinine values should be measured at least once a year	↑↑
12. In the first year of PTH replacement therapy, after reaching a stable state without dose adjustments, the therapy should be monitored quarterly and adjusted if necessary	↑↑

↑↑: 100% agreement, ↑: agreement > 85%

If treatment with calcium and active vitamin D is deemed inadequate, the Delphi participants unanimously recommend that subsequent PTH therapy should be monitored at least quarterly during the first year, after reaching a stable state without dose adjustments, and thereafter adjusted if necessary.

## Discussion

HypoPT is a rare condition, most commonly occurring following total thyroidectomy or neck dissection for head and neck tumors. Due to its rarity, detailed knowledge about its treatment is typically confined to specialized medical circles. However, HypoPT patients are often treated by non-specialists, such as general practitioners, making it essential to communicate consensus-based expert knowledge on diagnosis, treatment, and monitoring to these providers.

We identified five areas of common support that can offer valuable guidance to non-specialists managing HypoPT: Consensus has been reached on the etiology of the primary cause, which laboratory values to measure, when, and what other procedures can be used to ensure the diagnosis, and on treatment and monitoring (including how to do it, what goals to achieve, when, and how to review goals). Although the existing German and international guidelines already make recommendations, some of these are not specifically formulated and differ with regard to certain statements (Table 4).

**Table 4:** Guideline recommendations and further clarifications in this Delphi survey

Content of the existing guidelines	Clarification by this Delphi survey	Explanation for the clarification
Serum-calcium measurement within 24 h postoperative [21, 22, 27] In a case of hypocalcaemia and undetectable/low serum PTH levels, second measurement with at least 2 weeks apart, to confirm the diagnosis [22]	Serum albumin-adjusted calcium measurement within 24 h postoperatively useful, especially in the presence of HypoPT-specific symptoms Additional measurements of serum albumin-adjusted calcium within the first 2 postoperative weeks, then every 3 to 6 months	The existing German guideline is a surgical guideline and focuses on a short timeframe [27]. So far, measurements to predict which patients will not develop HypoPT are reported [22]. This work could help with clear recommendations for diagnosis
Defined threshold for a low serum PTH level of < 10 pg/mL [22]	No thresholds for a low serum PTH level reported	Due to the use of different generations of immunoassays that can result in different normal ranges of serum PTH concentrations, a universal threshold cannot be established, also see [31]
Cataract, infections, nephrocalcinosis/nephrolithiasis, renal insufficiency, seizures, depression, ischemic heart disease, arrhythmias are the most common complications [22] Various neuromuscular, CNS, cardiovascular, gastrointestinal, renal and respiratory symptoms associated with hypo- or hypercalcemia [21]	Paresthesia, muscle cramps, and fatigue are the three most common symptoms	Most important symptoms could be helpful for non-specialist physicians as diagnostic differentiation
Measurement of 24-h urine calcium and serum creatinine levels in a range of 6–24 months [22] Measurement of 24-h urine calcium and serum creatinine levels in a range of 3–6 months [21]	Measurement of 24-h urine calcium and serum creatinine levels once a year	The existing German guideline is a surgical guideline and focuses on a short timeframe [27]. In this context, long-term monitoring recommendations could help non-specialists in the follow-up
Using standardized questionnaires like SF-36 developed for chronic diseases and the "Hypoparathyroidism Patient Experience Scale (HPES) is a psychometrically validated, disease-specific measure specifically designed to assess the symptoms and impacts associated with hypoparathyroidism [22, 32] No instruments for QoL measurement mentioned [21, 27]	HR-QoL measurement by standardized questionnaires specially designed for HypoPT Naming of the HPQ28	So far, general quality of life questionnaires are recommended. For the situation of HypoPT patients, specific questionnaires as the HPQ28 could be helpful to assess quality of life
No information on monitoring of PTH replacement therapy [21, 22, 27], probably due to missing data	Therapy should be monitored quarterly in the first year of treatment after reaching a stable state	Monitoring intervals for patients who are being treated with PTH replacement therapy have been named that could help physicians to monitor these patients

Experts in the DACH region estimate that at least 90% of all cases of HypoPT are of postoperative nature. This estimate is at the upper end of the interval reported in the literature [2, 17–19].

For the diagnosis of HypoPT, serum albumin-adjusted calcium measurement is performed within the first 24 postoperative hours according to guidelines [21, 22, 27]. Low serum calcium is defined as a subnormal albumin-adjusted total calcium of ≤ 8.4 mg/dL or ≤ 2.1 mmol/L [33]. Khan et al. add that in the case of hypocalcemia and undetectable/low serum PTH levels, the diagnosis should be confirmed by a second measurement at least 2 weeks apart [22]. In addition to the established measurement time within the first 24 postoperative hours, German-speaking experts agree that additional measurements of serum calcium within the first 2 postoperative weeks and then every 3 to 6 months should be used. Consequently, measurement of serum calcium more than 24 h postoperatively is still useful, especially in the presence of HypoPT-specific symptoms.

These symptoms can be complex and vary between patients. Paresthesia, muscle cramps and fatigue, nephrocalcinosis/nephrolithiasis, seizures, depression, ischemic heart disease, arrhythmias and, in the long term, cataracts and renal insufficiency are just some of them [12, 13, 34]. The experts in this Delphi survey concluded that paresthesia, muscle cramps, and fatigue are the three most common symptoms. The measurement of 24-h urine calcium and serum creatinine are part of the long-term monitoring of patients with chronic HypoPT recommended by most international guidelines and aim to capture long-term renal complications of chronic HypoPT (nephrocalcinosis/nephrolithiasis) [21].

Other monitoring examinations such as kidney ultrasound, bone mineral density measurements and cranial CT scans are optional with higher significance in long-term disease and monitoring.

In addition to serum albumin-adjusted calcium, the measurement of serum PTH levels is the second key biochemical parameter for the diagnosis of HypoPT. There is a consensus in published guidelines and in our Delphi survey that a low serum PTH level is key for the diagnosis HypoPT. However, a defined threshold for a low serum PTH level of < 10 pg/mL in the immediate post-operative period is reported only by Khan et al. [22]. The Position Statement of the PARAT program identified an additional approach, namely calculating the ratio between pre- and post-surgical serum PTH concentrations and the decrease of > 70% as being strongly associated with chronic Hypo-PT [21].

The main problem in establishing serum PTH threshold levels is the use of different generations of immunoassays that result in different normal ranges of serum PTH concentrations [2]. This is supported by the meta-analysis of Nagel et al. [31]. Therein, 40 different PTH assays deriving from 81 studies were cited to avoid assay-related confusion in preoperative serum PTH and calcium measurements. They suggest that both serum PTH and albumin-adjusted calcium levels should be measured by the same laboratory where the initial postoperative measurements were performed [31]. For the diagnosis of HypoPT, the overall clinical manifestations in combination with low serum albumin-adjusted calcium and low serum PTH levels are more important diagnostic criteria for HypoPT than an isolated PTH value below a pre-defined threshold.

With regards to monitoring, a regular measurement of 24-h urine calcium and serum creatinine levels is widely used. Our experts agree that these parameters should be assessed once a year. Khan et al. [22] support a range of 6–24 months and Bollerslev et al. [21] recommend creatinine measurement every 3–6 months. In any case, measurement of the above values should be performed in a regular context within the specified period. Therefore, the results of this Delphi survey provide an operationalized guidance for these monitoring parameters.

Health-related quality of life is a key goal of treatment and monitoring. As this aspect is not reflected by biochemical measurements, further reference points are needed. The experts in this Delphi survey therefore unanimously agree on the importance of usage of standardized questionnaires specifically designed for HypoPT. While Bollerslev et al. [21] do not mention instruments to measure quality of life, Khan et al. [22] refer to monitoring using the standardized and validated “SF-36” [4] developed for chronic diseases and the “Hypoparathyroidism Patient Experience Scale (HPES) is a psychometrically validated, disease-specific measure specifically designed to assess the symptoms and impacts associated with hypoparathyroidism [32]. The SF-36 questionnaire, which was not explicitly developed for the HypoPT, is the most commonly used instrument for measuring quality of life in the literature [35]. Three HypoPT-specific questionnaires have been developed, the Hypoparathyroidism Symptom Diary [36], the [Hypoparathyroidism Patient Experience Scale (HPES) [32, 37], and the German disease-specific questionnaire HPQ28] [38–41]. Only the HPQ28 has been prospectively tested in HypoPT patients, validated compared to disease-specific control groups and the normal population and is available free of cost. The use of these specific questionnaires as part of the monitoring program of patients with chronic HypoPT, i.e., every 6–12 months, is recommended if possible.

No recommendations for possible PTH replacement therapies were included in the German postoperative guideline from 2021 [27]. Evaluation of PTH therapy is generally recommended for patients who are not well controlled on calcium and active vitamin D treatment. In the European Union, only two PTH therapies have been approved for the treatment of chronic HypoPT in adults (none in children). The first was a once-daily injection of rhPTH(1–84). As production ended in 2024, this treatment is no longer available [42]. The second is palopegteriparatide (or TransCon PTH), which is available as a once-daily injection. Palopegteriparatide is a prodrug of PTH(1–34), administered once-daily, designed to provide active PTH within the physiologic range for 24 h/day [43]. The primary goal of PTH-replacement therapy with palopegteriparatide is to maintain serum calcium within the normal range without need for additional treatment with active vitamin D and oral calcium beyond low doses (calcium dose ≤ 600 mg/d) [44]. This form of therapy therefore has the chance to significantly reduce daily pill burden of calcium and vitamin D, identified as one important factor decreasing quality of life in HypoPT patients [35, 45]

In the PaTHway study, independence from calcium and active vitamin D supplements was achieved in 79% of all participants receiving palopegteriparatide, and mean 24-h urine calcium was normalized. Furthermore, a significant treatment effect on wellbeing was seen, measured with the hypoparathyroidism patient experience scale (HPES) and 36-Item Short Form Survey (SF-36) [44]. When treating HypoPT with PTH replacement therapy, experts agree that therapy should be monitored quarterly after a stable dose is reached.

International guidelines agree on the use of PTH replacement therapy in selected patients who are not adequately controlled on therapy with calcium and active vitamin D supplementation. Khan et al. [22] recommend PTH replacement therapy in patients with symptomatic hypocalcemia, hyperphosphatemia, renal insufficiency, hypercalciuria, or poor quality of life. Bollerslev et al. [21] suggest PTH therapy for patients whose serum and urine calcium cannot be kept within target ranges in a stable and safe manner. Furthermore, serum albumin-adjusted calcium levels that are persistently outside the normal range and hypercalciuria are additional indications for PTH replacement therapy.

They also specify that PTH replacement therapy should be initiated in the event of persistent symptoms and/or the need for hospitalization, as well as in cases of poor tolerance of treatment with calcium and active vitamin D. This indicates that factors beyond biochemical parameters play a role in the response to treatment. This Delphi survey was conducted before and during the early phases of market entry of palopegteriparatide, therefore no wide spread experience with its usage in the German market existed during the time of this project.

While this Delphi survey provides valuable guidance from experts on HypoPT, there are several limitations to consider. First, the small sample size of seven clinical experts limits the generalizability of the findings. Although this number is consistent with the Delphi method, a larger panel might capture a broader spectrum of clinical opinions and practices.

Second, although we identified and reached consensus on many relevant factors for HypoPT diagnosis, treatment, and monitoring, this list is not exhaustive. Additional parameters and factors may be required in clinical practice depending on patient-specific needs and evolving treatment protocols. Future research should explore these additional parameters and validate them within broader patient populations to further enhance practical applicability.

Furthermore, while consensus was reached on many diagnostic and monitoring practices, areas such as the exact threshold for PTH levels and the optimal timing for identifying chronic HypoPT were less conclusive. This ambiguity highlights the ongoing need for research and further refinement of best practices for HypoPT management.

Lastly, while consensus was achieved on the importance of quality of life measurements, there is currently no universally accepted disease-specific tool for HypoPT in clinical practice. The HPQ28 questionnaire offers a promising approach being available in German and free of cost and should be progressively implemented in the regular care of HypoPT patients in future. Having been originally been drafted in German and English, it is now also available in other languages. In particular, it has been adapted and validated in French and Danish, while adaptations in Italian, Czech, Spanish and Turkish are pending. The still existing gap in QOL documentation underscores the importance of continued validation and implementation of such tools to better support patient-centered care in everyday clinical practice.

## Conclusion

The experts in this Delphi survey agree on a higher proportion of postoperative HypoPT rates in German-speaking countries compared to the proportions reported internationally, but no reliable data are available. According to the experts, the measurement of serum albumin-adjusted calcium for the diagnosis of HypoPT should be performed not only 24 h postoperatively, but also within 2 weeks postoperatively and then every 3 to 6 months. A later measurement, as described, should be performed by physicians especially if the overall clinical manifestations are suggestive of HypoPT. Paresthesia, muscle cramps, and fatigue were the most common symptoms reported by participants. The short-term goal of HypoPT treatment, in addition to achieving serum albumin-adjusted calcium levels in the lower normal range, is to improve health-related quality of life. To simplify and standardize the measurement of these, the clinical use of the freely available HPQ28, a German disease-specific validated questionnaire, which is currently being adapted and validated in other languages, would be useful in future. These additions to the existing guidelines can help general practitioners and other healthcare workers to better treat patients with HypoPT.

## Supplementary Information

Below is the link to the electronic supplementary material.

Supplementary file1 (DOCX 16 KB)

## Abbreviations

DACH: Germany, Austria, Switzerland
HypoPT: Hypoparathyroidism
PTH: Parathyroid hormone
rhPTH: Recombinant human parathyroid hormone

## References

[1] Petersenn S, Bojunga J, Brabant G et al (2019) Hypoparathyroidism—un underestimated problem? MMW Fortschr Med 161:12–20. 10.1007/s15006-019-1174-4 31828671 10.1007/s15006-019-1174-4

[2] Brandi ML, Bilezikian JP, Shoback D et al (2016) Management of hypoparathyroidism: summary statement and guidelines. J Clin Endocrinol Metab 101:2273–2283. 10.1210/jc.2015-3907 26943719 10.1210/jc.2015-3907

[3] Bilezikian JP, Brandi ML, Cusano NE et al (2016) Management of hypoparathyroidism: present and future. J Clin Endocrinol Metab 101:2313–2324. 10.1210/jc.2015-3910 26938200 10.1210/jc.2015-3910 PMC5393596

[4] Astor MC, Løvås K, Debowska A et al (2016) Epidemiology and health-related quality of life in hypoparathyroidism in Norway. J Clin Endocrinol Metab 101:3045–3053. 10.1210/jc.2016-1477 27186861 10.1210/jc.2016-1477 PMC4971340

[5] Cianferotti L, Parri S, Gronchi G et al (2018) Prevalence of chronic hypoparathyroidism in a Mediterranean region as estimated by the analysis of anonymous healthcare database. Calcif Tissue Int 103:144–150. 10.1007/s00223-018-0405-5 29516129 10.1007/s00223-018-0405-5

[6] Vadiveloo T, Donnan PT, Leese GP (2018) A Population-Based Study of the Epidemiology of Chronic Hypoparathyroidism. J Bone Miner Res 33:478–485. 10.1002/jbmr.3329 29087618 10.1002/jbmr.3329

[7] Clarke BL, Brown EM, Collins MT et al (2016) Epidemiology and diagnosis of hypoparathyroidism. J Clin Endocrinol Metab 101:2284–2299. 10.1210/jc.2015-3908 26943720 10.1210/jc.2015-3908 PMC5393595

[8] Verburg FA (2015) Is thyroid surgery performed too often in Germany? Nuklearmedizin 54:101–105 26105718

[9] Underbjerg L, Sikjaer T, Mosekilde L, Rejnmark L (2013) Cardiovascular and renal complications to postsurgical hypoparathyroidism: a Danish nationwide controlled historic follow-up study. J Bone Miner Res 28:2277–2285. 10.1002/jbmr.1979 23661265 10.1002/jbmr.1979

[10] Hari Kumar KVS, Patnaik SK (2017) Incidence of parathyroid disorders in Indian adult male population: a 25-year follow-up study. Clin Endocrinol (Oxf) 87:605–608. 10.1111/cen.13395 28617975 10.1111/cen.13395

[11] Yang Y-Y, Deng Y-H, Sun L-H et al (2025) Hypoparathyroidism: similarities and differences between Western and Eastern countries. Osteoporos Int 36:391–402. 10.1007/s00198-024-07352-6 39777494 10.1007/s00198-024-07352-6

[12] Siggelkow H (2017) Standardtherapie und Ausblick bei der Behandlung des Hypoparathyreoidismus. Endokrinologie Informationen 2(1):11–14

[13] Rejnmark L, Underbjerg L, Sikjaer T (2015) Hypoparathyroidism: replacement therapy with parathyroid hormone. Endocrinol Metab 30:436. 10.3803/EnM.2015.30.4.436 10.3803/EnM.2015.30.4.436 PMC4722396 26394728

[14] Yao L, Hui X, Li M et al (2022) Complications, symptoms, presurgical predictors in patients with chronic hypoparathyroidism: a systematic review. J Bone Miner Res 37:2642–2653. 10.1002/jbmr.4673 36375810 10.1002/jbmr.4673

[15] Bilezikian JP, Khan A, Potts JT et al (2011) Hypoparathyroidism in the adult: epidemiology, diagnosis, pathophysiology, target-organ involvement, treatment, and challenges for future research. J Bone Miner Res 26:2317–2337. 10.1002/jbmr.483 21812031 10.1002/jbmr.483 PMC3405491

[16] Shoback D (2008) Hypoparathyroidism. N Engl J Med 359:391–403. 10.1056/NEJMcp0803050 18650515 10.1056/NEJMcp0803050

[17] Pasieka JL, Wentworth K, Yeo CT et al (2022) Etiology and pathophysiology of hypoparathyroidism: a narrative review. J Bone Miner Res 37:2586–2601. 10.1002/jbmr.4714 36153665 10.1002/jbmr.4714 PMC10364481

[18] Khan AA, Koch CA, Van Uum S et al (2019) Standards of care for hypoparathyroidism in adults: a Canadian and international consensus. Eur J Endocrinol 180:P1–P22. 10.1530/EJE-18-0609 30540559 10.1530/EJE-18-0609 PMC6365672

[19] Kovaleva EV, Eremkina AK, Elfimova AR et al (2022) The Russian registry of chronic hypoparathyroidism. Front Endocrinol (Lausanne). 10.3389/fendo.2022.800119 35250859 10.3389/fendo.2022.800119 PMC8889095

[20] Roberts SL, El-Shikh M, Alam P, Borumandi F (2023) Incidence of post-surgical hypoparathyroidism (POSH) after total thyroidectomy. Br J Oral Maxillofac Surg 61:679–685. 10.1016/j.bjoms.2023.10.001 38126158 10.1016/j.bjoms.2023.10.001

[21] Bollerslev J, Rejnmark L, Zahn A et al (2022) European expert consensus on practical management of specific aspects of parathyroid disorders in adults and in pregnancy: recommendations of the ESE educational program of parathyroid disorders. Eur J Endocrinol 186:R33–R63. 10.1530/EJE-21-1044 34863037 10.1530/EJE-21-1044 PMC8789028

[22] Khan AA, Bilezikian JP, Brandi ML et al (2022) Evaluation and management of hypoparathyroidism summary statement and guidelines from the Second International Workshop. J Bone Miner Res 37:2568–2585. 10.1002/jbmr.4691 36054621 10.1002/jbmr.4691

[23] Ning K, Yu Y, Zheng X et al (2024) Risk factors of transient and permanent hypoparathyroidism after thyroidectomy: a systematic review and meta-analysis. Int J Surg 110:5047–5062. 10.1097/JS9.0000000000001475 38652139 10.1097/JS9.0000000000001475 PMC11326036

[24] Tsourdi E, Henneicke H, Fuss CT et al (2020) Novel treatment modalities in chronic hypoparathyroidism. Osteologie 29:283–292. 10.1055/a-1245-7539

[25] European Medicines Agency (EMA) (2023) Yorvipath: EPAR—product information. https://www.ema.europa.eu/en/documents/product-information/yorvipath-epar-product-information_en.pdf

[26] Marcucci G, Della Pepa G, Brandi ML (2016) Natpara for the treatment of hypoparathyroidism. Expert Opin Biol Ther 16:1417–1424. 10.1080/14712598.2016.1238455 27689826 10.1080/14712598.2016.1238455

[27] Deutsche Fachgesellschaft für Endokrinologie, Deutsche Gesellschaft für Nuklearmedizin, Deutsche Ges. für Kinderendkrinologie- und diabetologie (DGKED) e.V., et al (2012) S2k Leitlinie Operative Therapie benigner Schilddrüsenerkrankungen. https://register.awmf.org/assets/guidelines/088-007l_S2k_operative_Therapie_benigner_Schilddruesenerkrankungen_2025-03-verlaengert.pdf

[28] Humphrey-Murto S, Varpio L, Gonsalves C, Wood TJ (2017) Using consensus group methods such as Delphi and nominal group in medical education research. Med Teach 39:14–19. 10.1080/0142159X.2017.1245856 27841062 10.1080/0142159X.2017.1245856

[29] Attieh R, Gagnon M-P, Estabrooks CA et al (2014) Organizational readiness for knowledge translation in chronic care: a Delphi study. BMC Health Serv Res 14:534. 10.1186/s12913-014-0534-0 25380653 10.1186/s12913-014-0534-0 PMC4226850

[30] Christie CA, Barela E (2005) The delphi technique as a method for increasing inclusion in the evaluation process. Can J Program Eval 20:105–122. 10.3138/cjpe.020.005

[31] Nagel K, Hendricks A, Lenschow C et al (2022) Definition and diagnosis of postsurgical hypoparathyroidism after thyroid surgery: meta-analysis. BJS Open. 10.1093/bjsopen/zrac102 36050906 10.1093/bjsopen/zrac102 PMC9437325

[32] Brod M, Waldman LT, Smith A, Karpf D (2021) Living with hypoparathyroidism: development of the Hypoparathyroidism Patient Experience Scale-Impact (HPES-Impact). Qual Life Res 30:277–291. 10.1007/s11136-020-02607-1 32833143 10.1007/s11136-020-02607-1 PMC7847873

[33] Fischbach FT, Fischbach MA (2017) Fischbach’s A Manual of Laboratory and Diagnostic Tests, 10th edn. Lippincott Williams & Wilkins (LWW), Philadelphia

[34] Bilezikian JP (2020) Hypoparathyroidism. J Clin Endocrinol Metab 105:1722–1736. 10.1210/clinem/dgaa113 32322899 10.1210/clinem/dgaa113 PMC7176479

[35] Büttner M, Singer S, Taylor K (2024) Quality of life in patients with hypoparathyroidism receiving standard treatment: an updated systematic review. Endocrine 85:80–90. 10.1007/s12020-024-03807-2 38578400 10.1007/s12020-024-03807-2 PMC11246296

[36] Coles T, Chen K, Nelson L et al (2019) Psychometric evaluation of the hypoparathyroidism symptom diary. Patient Relat Outcome Meas 10:25–36. 10.2147/PROM.S179310 30774490 10.2147/PROM.S179310 PMC6357884

[37] Brod M, Waldman LT, Smith A, Karpf D (2020) Assessing the patient experience of hypoparathyroidism symptoms: development of the Hypoparathyroidism Patient Experience Scale-Symptom (HPES-Symptom). Patient 13:151–162. 10.1007/s40271-019-00388-5 31552607 10.1007/s40271-019-00388-5 PMC7075823

[38] Trummer C, Blaschke M, Quint D et al (2025) Normative values for the hypoparathyroidism patient questionnaire (HPQ28) in the German general population. J Patient Rep Outcomes 9:38. 10.1186/s41687-025-00868-3 40178746 10.1186/s41687-025-00868-3 PMC11968587

[39] Stamm B, Blaschke M, Wilken L et al (2022) The influence of conventional treatment on symptoms and complaints in patients with chronic postsurgical hypoparathyroidism. JBMR Plus 6:e10586. 10.1002/jbm4.10586 35229064 10.1002/jbm4.10586 PMC8861984

[40] Büttner M, Krogh D, Siggelkow H, Singer S (2023) Impairments in quality of life and predictors of symptom burden in patients with hypoparathyroidism: results from a population-based survey. Endocrine 82:419–426. 10.1007/s12020-023-03443-2 37450218 10.1007/s12020-023-03443-2 PMC10543843

[41] Wilde D, Wilken L, Stamm B et al (2019) The HPQ-development and first administration of a questionnaire for hypoparathyroid patients. JBMR Plus 4:e10245. 10.1002/jbm4.10245 31956849 10.1002/jbm4.10245 PMC6957982

[42] Rote Hand Brief Natpar (Parathyroidhormon) 100 Mikrogramm/Dosis Pulver und Lösungsmittel für Injektionslösung: voraussichtlicher Lieferengpass ab 1. Juli 2022. https://www.bfarm.de/SharedDocs/Risikoinformationen/Pharmakovigilanz/DE/RHB/2022/rhb-natpar_oktober2022.html

[43] Karpf DB, Pihl S, Mourya S et al (2020) A Randomized Double-Blind Placebo-Controlled First-In-Human Phase 1 Trial of TransCon PTH in Healthy Adults. J Bone Miner Res 35:1430–1440. 10.1002/jbmr.4016 32212275 10.1002/jbmr.4016 PMC9328939

[44] Khan AA, Rubin MR, Schwarz P et al (2022) Efficacy and safety of parathyroid hormone replacement with TransCon PTH in hypoparathyroidism: 26-week results from the phase 3 pathway trial. J Bone Miner Res 38:14–25. 10.1002/jbmr.4726 36271471 10.1002/jbmr.4726 PMC10099823

[45] Siggelkow H, Clarke BL, Germak J et al (2020) Burden of illness in not adequately controlled chronic hypoparathyroidism: findings from a 13-country patient and caregiver survey. Clin Endocrinol (Oxf) 92:159–168. 10.1111/cen.14128 31721256 10.1111/cen.14128 PMC7027891

