# Supplementary material for: Consensus-Based Recommendations for the Diagnosis, Treatment, and Monitoring of Hypoparathyroidism: Insights from the DACH Region
Source: Calcif Tissue Int. 2025 Aug 12;116(1):107. doi: 10.1007/s00223-025-01414-5 (PMC12343711; doi:10.1007/s00223-025-01414-5)
Supplement: Supplementary file 1 — Supplementary file1 (DOCX 16 KB) [file 223_2025_1414_MOESM1_ESM.docx]

**Consensus-Based Recommendations for the Diagnosis, Treatment, and Monitoring of Hypoparathyroidism: Insights from the DACH region**

Elena Tsourdi, Karin Amrein, Christian Meier, Markus Ketteler, Michael C. Kreissl, Annie Mathew, Tobias Vogelmann, Tino Schubert, Heide Siggelkow

**Supplemental Material**

| It is unlikely that a surgically induced hypoPT will recover without treatment after 6 months. | ↓ |
| --- | --- |
| PTH values should be determined quarterly after a successful neck operation | ↓ |
| On average, 50% of all HypoPT patients achieve their treatment goals | ↓ |
| Patient preference regarding oral therapies is the most important reason why patients who do not respond adequately to Calcium and active vitamin D are not switched to PTH therapy. | ↓ |
| Compliance concerns are the third most important reason why patients who do not respond adequately to calcium and active vitamin D are not switched to PTH therapy. | ↓ |
| Compliance problems are the most common reason for stopping PTH therapy. | ↓ |
| Patient preference regarding oral therapies is the second most common reason for discontinuing PTH therapy. | ↓ |
| Concerns about the appropriateness of therapy costs are the third most common reason for discontinuing PTH therapy. | ↓ |

Table S1: Statements without consensus reached

↓: no consensus reached
